# Fortune Favours the Bold: An Agent-Based Model Reveals Adaptive Advantages of Overconfidence in War

**DOI:** 10.1371/journal.pone.0020851

**Published:** 2011-06-24

**Authors:** Dominic D. P. Johnson, Nils B. Weidmann, Lars-Erik Cederman

**Affiliations:** 1 Politics and International Relations, University of Edinburgh, Edinburgh, United Kingdom; 2 Jackson Institute for Global Affairs, Yale University, New Haven, Connecticut, United States of America; 3 Centre for the Study of Civil War, International Peace Research Institute Oslo, Oslo, Norway; 4 International Conflict Research, Eidgenössische Technische Hochschule (ETH) Zurich, Zurich, Switzerland; University of Maribor, Slovenia

## Abstract

Overconfidence has long been considered a cause of war. Like other decision-making biases, overconfidence seems detrimental because it increases the frequency and costs of fighting. However, evolutionary biologists have proposed that overconfidence may also confer adaptive advantages: increasing ambition, resolve, persistence, bluffing opponents, and winning net payoffs from risky opportunities despite occasional failures. We report the results of an agent-based model of inter-state conflict, which allows us to evaluate the performance of different strategies in competition with each other. Counter-intuitively, we find that overconfident states predominate in the population at the expense of unbiased or underconfident states. Overconfident states win because: (1) they are more likely to accumulate resources from frequent attempts at conquest; (2) they are more likely to gang up on weak states, forcing victims to split their defences; and (3) when the decision threshold for attacking requires an overwhelming asymmetry of power, unbiased and underconfident states shirk many conflicts they are actually likely to win. These “adaptive advantages” of overconfidence may, via selection effects, learning, or evolved psychology, have spread and become entrenched among modern states, organizations and decision-makers. This would help to explain the frequent association of overconfidence and war, even if it no longer brings benefits today.

## Introduction

War is deemed a puzzle because if states were rational, they could avoid the costs of fighting in a pre-war bargain that reflected their relative power [1]. Of course, there are several caveats to such an expectation: strong states may anticipate the spoils of conquest [2], [3], and weak states may anticipate an improvement in their bargaining position, favourable intervention by third parties, or superior strategies and resolve [4], [5], [6], [7]. Nevertheless, war remains puzzling because it occurs even when these caveats are absent or unlikely, and both sides would be better off avoiding violence.

One solution to the war puzzle, long noted by historians and political scientists, is that people and states tend to be overconfident about their chances of success, reducing the perceived costs of war and increasing its perceived benefits [8], [9], [10], [11]. A general bias towards overconfidence has also been noted in economics [12], law [13], management [14], finance [15], and negotiation [16]. Indeed, the phenomenon of overconfidence is a standard result within the psychological literature, which finds that most normal people tend to exhibit cognitive and motivational biases exaggerating their capabilities, their illusion of control over events, and their perceived invulnerability to risk, collectively termed “positive illusions” [17], [18]. If anything, such individual level biases appear to be further exacerbated at the group, organizational and state levels [6], [19], [20], [21], and historical analyses suggest that states and organizations also frequently fail to update their behaviour given past failures [22], [23], [24]. Nobel Laureate Daniel Kahneman recently concluded that, of all the psychological biases he and his colleagues have uncovered over the last 40 years of the “cognitive revolution”, *all* of them promote hawkish decision-making [25].

There is growing support for a causal link between overconfidence and war: experimental war games found that overconfident individuals were more likely to make unprovoked attacks on their opponents [26]; recent case study analyses found that variation in confidence among state decision-makers during crises correlated with whether or not war broke out [8], [9], [11], [27]; and quantitative analyses of inter-state wars show that initiators have lost one-quarter to one-half of the wars they started since 1500 [28], and this has increased to a majority of wars since 1945 [7], [29].

The larger question that remains unresolved is *why* people exhibit a bias towards overconfidence in situations of conflict, given that overconfidence appears to invoke significant costs—preventing peaceful outcomes and increasing the frequency, expense, and risks of war (for example, by provoking more powerful opponents) [1], [9]. One possibility is that although overconfidence may sometimes lead to mistakes, on average and over time, overconfidence may in fact promote *advantageous* decisions, signals or behaviour after all (or may have done so in the past), in which case we would *expect* to observe it. Although they amount to systematic errors in assessment, positive illusions have been argued to serve the interests of those that hold them because they promote ambition, creativity, persistence, and performance in a variety of tasks and contexts [17]. Recently, it has specifically been suggested that positive illusions were favoured by natural selection because they were adaptive in conflict settings in our evolutionary past, serving to increase resolve and persistence [30], to bluff opponents [30], [31], [32] and, where the stakes are high enough, to exploit risky opportunities that generate a higher net pay off despite the costs of occasional failures [33], [34]. Of course, overconfidence can also be a conscious political tactic to boost morale, rally support, or deter rivals. But, as Robert Trivers has argued, an evolved psychological bias towards genuine, *self-deceptive* overconfidence would be especially effective because it generates more convincing beliefs and signals, reducing behavioural “leakage” from sham confidence that might give the game away to opponents [31], [32]. Whether overconfidence serves adaptive advantages in the setting of international conflict, however, remains an untested question.

## Methods

Since it is not possible to conduct real-world experiments on whether overconfidence is adaptive or not in international conflict, an alternative analytical tool is offered by agent-based models (ABMs) [35], [36]. ABMs have enjoyed increasing popularity in recent years as a way of exploring problems that remain beyond the reaches of experimental or analytical methods. Many such models have been inspired by evolutionary approaches [37], in which agents with different attributes compete with each other according to predefined behavioural rules. Successful strategies are replicated and spread while unsuccessful strategies die out, leading to evolutionary change in the proportions of each strategy in the population. ABMs have been applied to a wide range of problems, including studies of cooperation as well as conflict, identifying conditions that give rise, for example, to grouping behaviour [38], moralistic behaviour [39], cooperation in noisy conditions [40], and cooperation in spatial public goods games [41].

**Figure 1:**
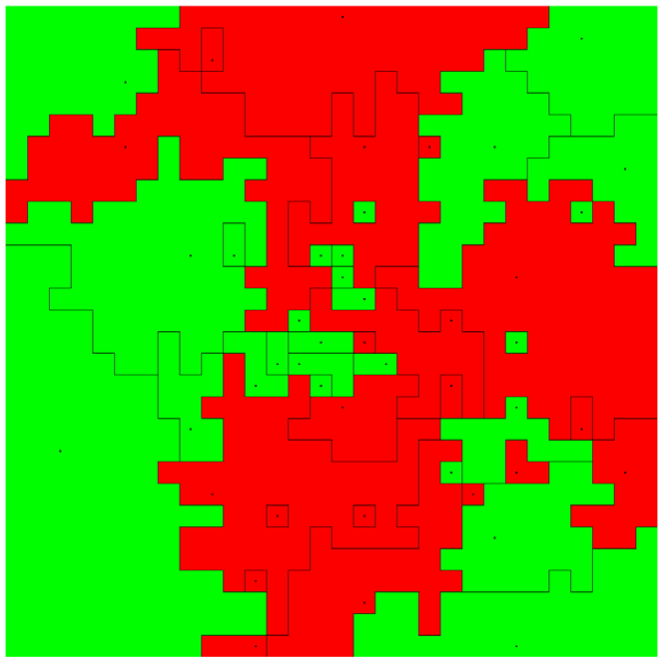
Initial starting conditions for a random simulation run, showing the simulation boundary (outer edges), state borders (black lines), and state capitals (black dots). Colours indicate different types of states (red: overconfident states, green: unbiased or underconfident states).

For our purposes, ABMs are useful because they allow us to compare the performance of overconfident states in competition with unbiased and underconfident states in a simulated spatial environment. Using a custom written ABM and following previously established protocols [35], [42], [43], we examined the relative performance of states in competition with each other on a 30×30 spatial grid (see Supporting Information for results with alternative parameter settings). The cells of the grid constitute “provinces”, and actors are represented by states of ≥1 contiguous provinces (see Figure 1). At each time step, states assess their neighbours and interact according to predefined decision-rules (described in full below). They attack if they identify a weaker opponent, and conflict outcomes are determined by a function of the warring states' relative resources (R). The process is then repeated over many generations.

We operationalize overconfidence by assigning each state a “confidence factor” α. A state's own *perceived* resource level is given by αR, so states with α>1 are overconfident, states with α = 1 are unbiased, and states with 0<α<1 are underconfident. While states with α≠1 distort the perception of their *own* strength, other states are not gullible and always see rivals' *true* strength. This is important for two reasons. First, the psychological literature on positive illusions shows that people tend to overestimate their *own* capabilities and prospects, but people are *not* biased when evaluating the attributes of third parties [17], [18], [44]. Second, if other states were gullible and believed the overconfident claims of aggressors, they would simply back off in the face of a bluff and concede, making overconfidence automatically advantageous; obviously we did not want to prime the model towards this trivial outcome.

αR is only used in deciding *whether or not to fight*. Actual R is used in determining war *outcomes*. Initial α values are randomly drawn from a log-normal distribution, which bounds values at zero but allows some states to have very high levels of overconfidence (this mimics reality: values less than zero are meaningless, while the long positive tail allows for a few very overconfident states). With an underlying μ = 0 and σ = 1, this distribution means that simulations begin with a population that is unbiased as a whole, with median α = 1.

For each of the initial *N* states (default *N* = 50), one province is chosen to host the capital. In each time step, all states synchronously execute 5 sub-procedures [35], [42]: (1) *resource extraction phase*—the state extracts one unit of resources from each of its provinces and adds it to its current resource level (all states begin life with 10 units per province); (2) *decision phase*—states assess the probability *p* of defeating each neighbour, and attack the state conferring the highest *p* (as long as *p* exceeds a given “attack threshold” *w*, default *w* = 0.5); (3) *resource-allocation phase*—states divide resources among all “active fronts” (wars with neighbouring states, whoever initiated them) in proportion to the size of each of those states; (4) *interaction phase*—war outcomes are determined by a logistic conflict success function (CSF) [45]:
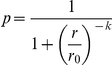
in which the likelihood *p* of winning for an attacker depends on the share *r* of its own resources R_A_ allocated to the fight, compared with the total resources *r*_0_ allocated to the fight by both the attacking and target state (R_A_+R_T_). *r*_0_ controls the proportion of resources at which the odds of winning are equal, and is kept constant at 0.5 throughout all simulations. A slope parameter *k* determines the decisiveness of conflict (i.e., how much resource differentials influence outcomes; default *k* = 5). The CSF is public information, so when making decisions about whether or not to fight, states can compute their true probability of defeating an opponent with a given resource level (although a states' *perceived* resources may differ from their *actual* resources, if α≠1). The only thing that states cannot anticipate is whether some of their own or their opponent's resources will have to be diverted elsewhere (if they, and/or their opponent, are attacked by a third party and thereby forced to open up a second front in that same time step). This uncertainty is, however, quite realistic (consider the uncertainty about many states' intentions prior to 1914, the US in 1917, Britain in 1938, or Russia, Japan, and the US in 1941). Finally, (5) *structural change phase*—the winning state gains a randomly selected adjacent province from the loser's territory. Provinces become independent states if their capital is: (1) captured; or (2) geographically cut off [35]. Such “newborn” states inherit the strategy (α) of their former state. No other processes of geopolitical change, such as secession of a subset of a state, can occur in the current model.

**Figure 2:**
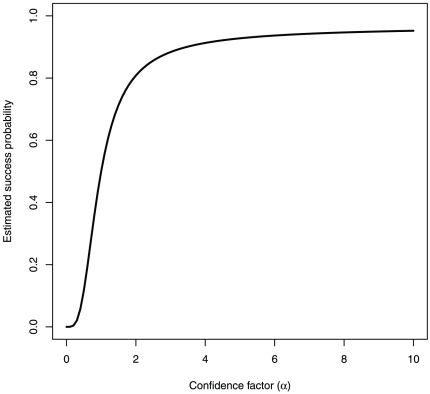
Estimated success probability for various confidence factors (α) in a hypothetical conflict between an attacker and target of equal strength (R_A_ = R_T_). The curve shows how the estimated probability of success for actor A changes with its confidence factor α. When α = 1 (signifying an unbiased actor), state A assesses its chances of winning correctly as 0.5. Overconfident states (α>1) overestimate their true probability of winning, and underconfident states (α<1) underestimate their true probability of winning, with asymptotes of winning probability at 0 and 1.

Overconfident states overestimate their relative resources (and hence their chances of winning wars against other states, see Figure 2), and will therefore attack more frequently since they perceive more viable targets. This means that overconfident actors should do worse than unbiased or underconfident states because they fight *extra* wars, and these extra wars will always be *against stronger states*, which they will tend to lose. The null hypothesis, then, is that overconfident states should be wiped out of the population. However, does this prediction hold in an *n*-player, spatial setting?

## Results

**Figure 3:**
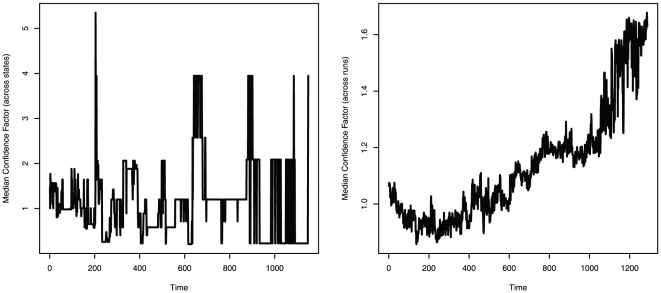
Results of simulations showing how the confidence of surviving states changes over time. Panel A shows a single example simulation run, with a typical pattern that the median confidence factor undergoes significant variation over time but stabilizes at a level well above 1. In this case, the strategy that comes to predominate is one that overestimates its resources by a factor of around 4. Panel B shows median confidence factors aggregated over 200 runs of the simulation. Simulations were terminated if there were 50 time steps with no fighting, or if only one state was left. In the majority of cases, only one state remained.

In stark contrast to the prediction, overconfidence consistently emerges as the predominant strategy in the model (see Figure 3; Movie S1 in the Supporting Information shows an example of a single simulation run). This result is robust to large changes in model parameters (e.g. size of the grid, whether it has boundaries or is a continuous surface, initial polarity, *k*, and distribution of α; see Supporting Information Table S1). However, the best performing states have a middling level of overconfidence: states with extremely high or low α do not perform well, suggesting that there is an “optimal margin of illusion”, as has been suggested in the psychology literature [46]. The superior performance of overconfident states can be attributed to three different phenomena, discussed in turn below: the “lottery effect”, offensive alliances, and attack thresholds.

### The “lottery effect”

We call the first phenomenon the *lottery effect*. Even though overconfident states are expected to lose more wars, they also enter more wars than unbiased or underconfident states—effectively “buying more lottery tickets” in the competition for survival. Many of these overconfident states will overreach themselves and be destroyed, but others will, by chance, enjoy consecutive victories and expand quickly. Overconfident states—and their associated high levels of α—are therefore more likely to be represented among the states that survive than are unbiased or underconfident states. If this seems counter-intuitive, note that all states—even overconfident ones—maximize *p* (the probability of defeating an opponent) in deciding which neighbour to attack (see Methods), so aggressive states tend to choose weaker targets rather than stronger targets just like any other state. However, because they pick more fights overall than anyone else, overconfident states have the best chance of gaining new territory and expanding. By contrast, underconfident or unbiased states are more likely to fight only when they are victims of attack by stronger neighbours and therefore tend to be destroyed by gradual attrition. Overconfident states also benefit from positive feedback: states that gain an early size advantage enjoy increased resources (R), which compounds their advantage as they are increasingly likely to win subsequent wars as a result, as well as accruing relative gains by denying rivals finite resources with which to compete in the future (akin to the advantages of “spiteful” territorial expansion among animals proposed by Verner [47]).

### Offensive alliances

The second reason for the success of overconfident states is that their targets will often be the simultaneous targets of other neighbouring states in the same time step (being the weakest in the vicinity). Because such a victim has to split its defensive resources to fight both (or more) attackers, the probability of defeating the weak state is increased even further. Since overconfident states are more likely to attack other states in the first place, they are also more likely to benefit from this effect. Thus, there can be offensive “alliances” that emerge automatically in the model (without any cooperation or planning). Obviously, if the model were contrived to allow *defensive* alliances, these could help to protect weak states and decrease the advantage of overconfident states. However, what is interesting is that the model shows that offensive alliances can emerge spontaneously on their own, whereas defensive alliances cannot (they would need higher level mechanisms of coordination and commitment to solve the problem of collective action and preventing free-riders).

### Attack Thresholds

Another important influence on the relative success of alternative strategies is the attack threshold (*w*)—the power asymmetry required for states to attack another. All our simulations presented above set the attack threshold at 0.5 (meaning that unbiased states only attack if the odds are in their favour). However, altering *w* has an important effect on optimum levels of confidence. If *w*<0.5, then *underconfident* states are favoured (notwithstanding some persistence of the other phenomena outlined above), because both unbiased and overconfident states would increase their frequency of attacks against stronger states that they are likely to lose against. This would give rise to an especially peaceful world dominated by cautious states. By contrast, if *w*>0.5, then *overconfident* states are favoured—even more than they already were in the simulations presented above—because both underconfident and unbiased states would decrease their frequency of attacks against states that they are actually likely to defeat. This would give rise to an especially war prone world dominated by belligerent states. Although overconfidence may be a dangerous strategy for any one state (many such states die), in a world in which wars tend not to happen unless one state has an overwhelming power advantage (*w*>0.5), it is an overconfident state that is likely to become king. Because of this logic, increasing *w* makes overconfidence a *more* successful strategy than it already is. This effect is ironic, because it means that in a world in which states are *reluctant* to attack without a significant power advantage (*w*>0.5; which is arguably closer to the real world than the other way around [48], [49]), the states that come to dominate are, paradoxically, more likely to be overconfident (akin to the United States being certain it could defeat North Vietnam or Iraq, but underestimating the costs nevertheless).

### Overconfidence versus aggression and risk-taking

Note that the key behavioural difference between overconfident actors and other actors was that they were more likely to attack a given opponent. This suggests that anything that increases *aggressiveness* or *risk-taking*—rather than overconfidence—could be advantageous. However, the decision-mechanism leading to overconfidence (and as specified in our model) differs in important ways from these alternatives.

Aggression implies a willingness to attack whether you believe you will win or not. Risk-taking implies a willingness to attack despite a (recognized) low probability of winning. Overconfident states do not fit either description—they only attack when they believe they will win. Overconfidence therefore represents a very different assessment and decision-making mechanism from aggression and risk-taking. Our model focuses specifically on overconfidence, operationalized as states believing they are stronger than they are in reality, and this bias directly influences their decision to fight a given opponent or not.

This leads to a related but separate question. If a propensity to attack other states is advantageous, as it was in our simulations, then is aggression, risk-taking, or overconfidence the best *means* to achieve this behaviour? We suggest that overconfidence may offer the best proximate mechanism than either aggression or risk-taking, for two reasons. First, overconfidence does take probabilities of winning into account, even if they are somewhat distorted, and this will lead to fewer defeats than a pure aggression strategy which ignores them. Second, overconfidence relies on a very simple rule: overestimating one's strength by a fixed amount. This avoids the need for extensive and accurate information about true capabilities and probabilities of all actors and outcomes typically required of a risk preference approach. Overconfidence is bounded, efficient, and fast—considerations that may have been particularly important if it emerged though an evolutionary process.

## Discussion

Contrary to intuition, a bias towards overconfidence can be an advantageous strategy. Despite wide variations in the basic parameters of our model, overconfident states consistently came to predominate over time at the expense of unbiased and underconfident competitors. The extent to which overconfidence pays off may actually be rather conservative in our model because, in the real world, believing or signalling that one has exaggerated strength through overconfidence can also serve to: (1) deter rivals [30], [31]; (2) attract allies; (3) extract greater concessions in bargaining; (4) increase resolve [17], ; (5) hedge against worse errors [33], [34]; (6) garner public support for war; and (7) win elections. None of these effects are included in our model, but all suggest additional mechanisms by which overconfidence can lead to adaptive advantages.

Overconfidence may seem an implausible strategy because it violates conventional formulations of rationality [1]. However, the appropriate metric of success in competitive situations is “ecological rationality”—the strategy that best exploits the prevailing environment, whatever that strategy may be [37], [50]. It is also important to recognize that overconfidence can spread via more than one mechanism. For example, if the *strategy* of overconfidence represents an ideology (akin to a gene), and the states represent the *entities* that carry these ideologies (akin to an individual organism) then, as in conventional natural selection, overconfidence as a strategy can spread even if it causes many of its bearers to die [51]. This is supported by recent modelling which suggests that a trait for risking death in war could arise through cultural group selection where there is strong inter-group competition [52], [53].

**Figure 4:**
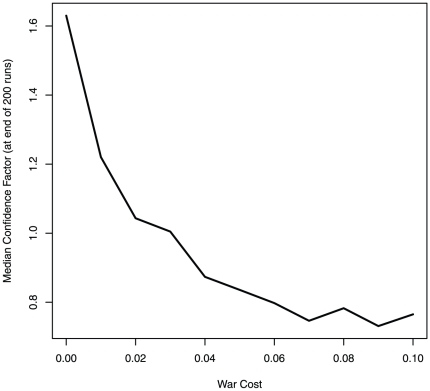
Median confidence factor at the end of the simulation decreases as war costs increase (line represents the median of 200 simulations for each level of war costs). Overconfident states prevail in the population as long as war costs are relatively low. As war costs increase they counteract the favourable effect of overconfidence. Note, however, that this decrease would be offset by altering other settings of the model, such as increasing the attack threshold, *w*, above 0.5, or allowing the spoils of victory to exceed the losses of defeat, instead of the current setup in which there is a zero-sum gain or loss of a province.

Overconfidence is not the best strategy under all conditions. We have already noted that unbiased or underconfident strategies would do better if *w*<0.5. To further examine the constraints of overconfidence, we added war costs *c*, making violent interaction between states increasingly expensive. As well as the gains and losses from the *outcomes* of war (the win/loss of a province, the latter of which already represented a cost of war within our model, but was limited to the loser only), the act of fighting now inflicts a given damage to each opponent, which is deducted from its resource level. War costs for a given state A are determined as a share *q* of the resources invested in the conflict by its opponent (the target state, T): *c*_A_ = *q* * R_T_, 0<*q*<1. As before, αR_A_ determines decisions for war, while R_A_ determines war outcomes. Clearly, and unsurprisingly, there are limits on the advantages of overconfidence as the costs of fighting increase (see Figure 4). While this may appear to undermine the adaptive advantages of overconfidence in war, note that: (1) overconfidence can remain the predominant strategy even when war is costly, up to a point; (2) overconfidence would remain the predominant strategy over a larger range of war costs if the attack threshold is increased (*w*>0.5); and (3) importantly, conflict in our model was always assumed to be zero-sum (one state wins 1 unit of territory at the expense of the opponent losing that 1 unit). In the real world, conflicts are often fought over *non*-zero-sum stakes, such as resources or land that neither actor owns in the first place. As the ratio of the value of the prize increases relative to the costs incurred in trying to obtain it, again overconfidence would become the predominant strategy over a larger range of war costs. Finally, note that political leaders—those making the decisions for war—do not always expect or personally experience any costs of war, even though they tend to reap its spoils. It is therefore unclear whether the costs of war would necessarily impact on the selection of overconfident traits at all, let alone succeed in counteracting evolved psychological propensities towards overconfidence in the modern world.

Do the advantages of overconfidence identified in our model have real-world empirical validity? The lottery effect certainly has some real-world analogues. Historically, successful conquerors are typically those that made aggressive moves to gain early footholds, which solidified their own position and disadvantaged rivals, as exemplified in the so-called “scramble for Africa” and the conquest of the Indian subcontinent [54], [55]. Moreover, in international relations theory, Stephen Van Evera cites as one of the four main causes of war the situation in which states see the chance to gain some resource that will facilitate future cumulative gains later on [9].

The power of offensive alliances also has real-world analogues. States and political elites have historically found it easy to combine forces where there is an opportunity to exploit weak rivals, while the converse of forming defensive alliances to help others in danger is extremely hard and suffers from an intense free-rider problem [56], [57]. Although defensive alliances have commonly occurred in the face of great mutual threats (such as against Germany in WW II), they depend on complex agreements or treaties that require considerable coordination and credible signals of commitment. States—especially weak states—often have a greater incentive to “bandwagon” with a powerful aggressor rather than taking the risk of “balancing” against them, since others may defect on such collective action leaving a balancer exposed and vulnerable. International relations theorists have also noted an interaction with the relative advantages of offense and defense. Perceived or actual defensive advantages (where *w*>0.5) leads to more free-riding and the avoidance of defensive alliances [58]—precisely the kind of world in which we expect overconfidence to spread.

Our model obviously lacks many aspects of realism—for example, states may not always be searching for opportunities to attack each other at every turn, and we do not allow states to learn from their own experience, or from observing others. Allowing periods of peace or learning in the model, however, would not change our results. Reducing the frequency of war would simply slow down the model, but does not alter the differential advantages of alternative strategies when wars occur. Conversely, allowing states to copy the strategies of successful competitors would simply speed up the model, since states would learn that overconfidence is the winning strategy and this would be rapidly copied from state to state rather than having to wait for the selection effects of pre-existing overconfident states to spread, one province at a time. Finally, as discussed above, in the real world expansionist states tend to be balanced by alliances of other powers [57]. However, the interesting thing about this is that offensive alliances can emerge automatically in our model whereas defensive alliances cannot. Defensive alliances require special conditions to be added ex ante. International security regimes such as NATO arise precisely because of the danger of expansionist states and the inability of weak targets to protect themselves without binding prior commitments to solve the collective action problem.

Our model suggests that the broad macro-historical process of inter-state competition may have selected for overconfidence, simply because it was—at some point—a successful strategy in competition with alternative strategies. However, while overconfidence may have been adaptive state behaviour in historical contexts, we do not claim that it remains adaptive behaviour today. While states and their ruling elites may have reaped great personal or national benefits from war until recent times [2], [3], and/or *learned* that bellicosity is an effective strategy (from their own or other states' histories), wars in the 21^st^ century have increasing domestic, international, and economic costs—low casualty tolerance, norms against conquest, legal responsibilities, intervention by collectives such as the UN or NATO, and the great resolve of nationalist insurgencies [7], [20], [59], [60], [61]. It is therefore likely that, today, overconfidence does nothing more than hinder political adjustment to the increasing costs of modern war. Unfortunately, given a deeply rooted human psychology that tends to bias decision-making towards hawkish behaviour [25], [30], overconfidence is likely to remain a prevalent political phenomenon, even if it causes considerable death and destruction for little gain.

## Supporting Information

Movie S1 **Quicktime movie of a single simulation run as displayed in** **Figure 3a** **, showing the actual step-by-step interactions of states on the grid.**
(MP4)

Table S1 **Simulation results using alternative parameter settings. As reported in the main paper, overconfidence consistently emerged as the dominant strategy in our agent-based model, as long as war costs remained relatively low.** Here we show that the predominance of overconfidence is robust to large changes in model parameters. The table reports the results of simulations with all combinations of the following alternative parameter values: (1) the size of the grid (20×20, 30×30, or 40×40); (2) whether the grid was a finite square with borders, or a continuous wrap-around Torus with no borders (yes/no); (3) the initial polarity (number of states) on the grid (10, 50, or 100); (4) the decisiveness of conflict, *k* (3, 5, or 7); and (5) the standard deviation of the initial distribution of confidence factors, α (0.5, 1.0, or 1.5). The final column in each row displays the median confidence after 50 runs with different random seeds. Each individual run continued until one of two termination criteria occurred: (1) 50 time steps with no fighting; or (2) only one state was left. In the majority of cases, only one state remained. In all cases, the median confidence factor at the end of the simulation was greater than 1.0, corresponding to the predominance of the overconfident strategy (for all the simulation results in the table, summary statistics for median confidence factors are: mean = 1.611, standard deviation = 0.467, range 1.047–3.091). In all cases reported here, war costs were zero (see main text for the effects of war costs), and the initial confidence parameter distribution was set to a mean of zero (which corresponds to an unbiased population on average).
(DOC)
