# Supplementary material for: Fortune Favours the Bold: An Agent-Based Model Reveals Adaptive Advantages of Overconfidence in War
Source: PLoS One. 2011 Jun 24;6(6):e20851. doi: 10.1371/journal.pone.0020851 (PMC3123293; doi:10.1371/journal.pone.0020851)
Supplement: Table S1 — Simulation results using alternative parameter settings. As reported in the main paper, overconfidence consistently emerged as the dominant strategy in our agent-based model, as long as war costs remained relatively low. Here we show that the predominance of overconfidence is robust to large changes in model parameters. The table reports the results of simulations with all combinations of the following alternative parameter values: (1) the size of the grid (20×20, 30×30, or 40×40); (2) whether the grid was a finite square with borders, or a continuous wrap-around Torus with no borders (yes/no); (3) the initial polarity (number of states) on the grid (10, 50, or 100); (4) the decisiveness of conflict, k (3, 5, or 7); and (5) the standard deviation of the initial distribution of confidence factors, α (0.5, 1.0, or 1.5). The final column in each row displays the median confidence after 50 runs with different random seeds. Each individual run continued until one of two termination criteria occurred: (1) 50 time steps with no fighting; or (2) only one state was left. In the majority of cases, only one state remained. In all cases, the median confidence factor at the end of the simulation was greater than 1.0, corresponding to the predominance of the overconfident strategy (for all the simulation results in the table, summary statistics for median confidence factors are: mean = 1.611, standard deviation = 0.467, range 1.047–3.091). In all cases reported here, war costs were zero (see main text for the effects of war costs), and the initial confidence parameter distribution was set to a mean of zero (which corresponds to an unbiased population on average). (DOC) [file pone.0020851.s002.doc]

**Table S1.**

| Grid Size | Initial Polarity | Torus | k | Standard Deviation | Median confidence |
| --- | --- | --- | --- | --- | --- |
| 20 | 10 | No | 3 | 0.5 | 1.078288 |
| 20 | 10 | Yes | 3 | 0.5 | 1.046867 |
| 20 | 10 | No | 3 | 1 | 1.238578 |
| 20 | 10 | Yes | 3 | 1 | 1.148775 |
| 20 | 10 | No | 3 | 1.5 | 1.295906 |
| 20 | 10 | Yes | 3 | 1.5 | 1.272346 |
| 20 | 10 | No | 5 | 0.5 | 1.1064 |
| 20 | 10 | Yes | 5 | 0.5 | 1.075624 |
| 20 | 10 | No | 5 | 1 | 1.275382 |
| 20 | 10 | Yes | 5 | 1 | 1.156986 |
| 20 | 10 | No | 5 | 1.5 | 1.378496 |
| 20 | 10 | Yes | 5 | 1.5 | 1.342608 |
| 20 | 10 | No | 7 | 0.5 | 1.112897 |
| 20 | 10 | Yes | 7 | 0.5 | 1.083587 |
| 20 | 10 | No | 7 | 1 | 1.275382 |
| 20 | 10 | Yes | 7 | 1 | 1.217016 |
| 20 | 10 | No | 7 | 1.5 | 1.627107 |
| 20 | 10 | Yes | 7 | 1.5 | 1.48485 |
| 20 | 50 | No | 3 | 0.5 | 1.166214 |
| 20 | 50 | Yes | 3 | 0.5 | 1.115519 |
| 20 | 50 | No | 3 | 1 | 1.745439 |
| 20 | 50 | Yes | 3 | 1 | 1.575788 |
| 20 | 50 | No | 3 | 1.5 | 2.121467 |
| 20 | 50 | Yes | 3 | 1.5 | 1.866561 |
| 20 | 50 | No | 5 | 0.5 | 1.214108 |
| 20 | 50 | Yes | 5 | 0.5 | 1.198276 |
| 20 | 50 | No | 5 | 1 | 1.666694 |
| 20 | 50 | Yes | 5 | 1 | 1.47615 |
| 20 | 50 | No | 5 | 1.5 | 1.794153 |
| 20 | 50 | Yes | 5 | 1.5 | 2.584218 |
| 20 | 50 | No | 7 | 0.5 | 1.304851 |
| 20 | 50 | Yes | 7 | 0.5 | 1.348982 |
| 20 | 50 | No | 7 | 1 | 1.65104 |
| 20 | 50 | Yes | 7 | 1 | 2.14729 |
| 20 | 50 | No | 7 | 1.5 | 2.205833 |
| 20 | 50 | Yes | 7 | 1.5 | 2.152524 |
| 20 | 100 | No | 3 | 0.5 | 1.176171 |
| 20 | 100 | Yes | 3 | 0.5 | 1.213444 |
| 20 | 100 | No | 3 | 1 | 1.624357 |
| 20 | 100 | Yes | 3 | 1 | 1.519211 |
| 20 | 100 | No | 3 | 1.5 | 2.084683 |
| 20 | 100 | Yes | 3 | 1.5 | 2.112545 |
| 20 | 100 | No | 5 | 0.5 | 1.320253 |
| 20 | 100 | Yes | 5 | 0.5 | 1.197655 |
| 20 | 100 | No | 5 | 1 | 1.743095 |
| 20 | 100 | Yes | 5 | 1 | 1.723866 |
| 20 | 100 | No | 5 | 1.5 | 2.028125 |
| 20 | 100 | Yes | 5 | 1.5 | 1.520496 |
| 20 | 100 | No | 7 | 0.5 | 1.321023 |
| 20 | 100 | Yes | 7 | 0.5 | 1.261322 |
| 20 | 100 | No | 7 | 1 | 1.977384 |
| 20 | 100 | Yes | 7 | 1 | 1.491917 |
| 20 | 100 | No | 7 | 1.5 | 3.029502 |
| 20 | 100 | Yes | 7 | 1.5 | 2.489041 |
| 30 | 10 | No | 3 | 0.5 | 1.142339 |
| 30 | 10 | Yes | 3 | 0.5 | 1.071809 |
| 30 | 10 | No | 3 | 1 | 1.28673 |
| 30 | 10 | Yes | 3 | 1 | 1.254571 |
| 30 | 10 | No | 3 | 1.5 | 1.607808 |
| 30 | 10 | Yes | 3 | 1.5 | 1.256881 |
| 30 | 10 | No | 5 | 0.5 | 1.162254 |
| 30 | 10 | Yes | 5 | 0.5 | 1.162254 |
| 30 | 10 | No | 5 | 1 | 1.293364 |
| 30 | 10 | Yes | 5 | 1 | 1.23027 |
| 30 | 10 | No | 5 | 1.5 | 1.471003 |
| 30 | 10 | Yes | 5 | 1.5 | 1.36459 |
| 30 | 10 | No | 7 | 0.5 | 1.142339 |
| 30 | 10 | Yes | 7 | 0.5 | 1.097295 |
| 30 | 10 | No | 7 | 1 | 1.351125 |
| 30 | 10 | Yes | 7 | 1 | 1.23027 |
| 30 | 10 | No | 7 | 1.5 | 1.538863 |
| 30 | 10 | Yes | 7 | 1.5 | 1.571023 |
| 30 | 50 | No | 3 | 0.5 | 1.193741 |
| 30 | 50 | Yes | 3 | 0.5 | 1.087871 |
| 30 | 50 | No | 3 | 1 | 1.488703 |
| 30 | 50 | Yes | 3 | 1 | 1.420167 |
| 30 | 50 | No | 3 | 1.5 | 1.962727 |
| 30 | 50 | Yes | 3 | 1.5 | 2.102057 |
| 30 | 50 | No | 5 | 0.5 | 1.225544 |
| 30 | 50 | Yes | 5 | 0.5 | 1.238531 |
| 30 | 50 | No | 5 | 1 | 1.585633 |
| 30 | 50 | Yes | 5 | 1 | 2.038252 |
| 30 | 50 | No | 5 | 1.5 | 2.493844 |
| 30 | 50 | Yes | 5 | 1.5 | 1.938964 |
| 30 | 50 | No | 7 | 0.5 | 1.251374 |
| 30 | 50 | Yes | 7 | 0.5 | 1.238531 |
| 30 | 50 | No | 7 | 1 | 1.707024 |
| 30 | 50 | Yes | 7 | 1 | 1.815002 |
| 30 | 50 | No | 7 | 1.5 | 2.692112 |
| 30 | 50 | Yes | 7 | 1.5 | 2.440489 |
| 30 | 100 | No | 3 | 0.5 | 1.33607 |
| 30 | 100 | Yes | 3 | 0.5 | 1.242623 |
| 30 | 100 | No | 3 | 1 | 1.662389 |
| 30 | 100 | Yes | 3 | 1 | 1.809222 |
| 30 | 100 | No | 3 | 1.5 | 2.635684 |
| 30 | 100 | Yes | 3 | 1.5 | 2.330837 |
| 30 | 100 | No | 5 | 0.5 | 1.546711 |
| 30 | 100 | Yes | 5 | 0.5 | 1.306295 |
| 30 | 100 | No | 5 | 1 | 2.155232 |
| 30 | 100 | Yes | 5 | 1 | 2.105559 |
| 30 | 100 | No | 5 | 1.5 | 2.745962 |
| 30 | 100 | Yes | 5 | 1.5 | 2.528309 |
| 30 | 100 | No | 7 | 0.5 | 1.483908 |
| 30 | 100 | Yes | 7 | 0.5 | 1.432371 |
| 30 | 100 | No | 7 | 1 | 1.830456 |
| 30 | 100 | Yes | 7 | 1 | 1.79011 |
| 30 | 100 | No | 7 | 1.5 | 3.091147 |
| 30 | 100 | Yes | 7 | 1.5 | 2.179993 |
| 40 | 10 | No | 3 | 0.5 | 1.197931 |
| 40 | 10 | Yes | 3 | 0.5 | 1.28525 |
| 40 | 10 | No | 3 | 1 | 1.392352 |
| 40 | 10 | Yes | 3 | 1 | 1.713365 |
| 40 | 10 | No | 3 | 1.5 | 1.991898 |
| 40 | 10 | Yes | 3 | 1.5 | 2.242724 |
| 40 | 10 | No | 5 | 0.5 | 1.166263 |
| 40 | 10 | Yes | 5 | 0.5 | 1.341962 |
| 40 | 10 | No | 5 | 1 | 1.453593 |
| 40 | 10 | Yes | 5 | 1 | 1.559709 |
| 40 | 10 | No | 5 | 1.5 | 1.752525 |
| 40 | 10 | Yes | 5 | 1.5 | 2.336336 |
| 40 | 10 | No | 7 | 0.5 | 1.179928 |
| 40 | 10 | Yes | 7 | 0.5 | 1.262123 |
| 40 | 10 | No | 7 | 1 | 1.453593 |
| 40 | 10 | Yes | 7 | 1 | 1.652506 |
| 40 | 10 | No | 7 | 1.5 | 1.822634 |
| 40 | 10 | Yes | 7 | 1.5 | 2.336336 |
| 40 | 50 | No | 3 | 0.5 | 1.272296 |
| 40 | 50 | Yes | 3 | 0.5 | 1.401449 |
| 40 | 50 | No | 3 | 1 | 1.880391 |
| 40 | 50 | Yes | 3 | 1 | 1.975357 |
| 40 | 50 | No | 3 | 1.5 | 2.060407 |
| 40 | 50 | Yes | 3 | 1.5 | 2.556082 |
| 40 | 50 | No | 5 | 0.5 | 1.287595 |
